# Beyond the Expectation: Pembrolizumab-Associated Tumor Lysis Syndrome in Metastatic Gastric Adenocarcinoma

**DOI:** 10.7759/cureus.48024

**Published:** 2023-10-31

**Authors:** Arwa Battah, Iyad Farouji, Theodore R DaCosta, Darshan Ghandi, Theodore DaCosta, Yatinder Bains

**Affiliations:** 1 Internal Medicine, New York Medical College at Saint Michael's Medical Center, Newark, USA; 2 Internal Medicine, Saint Marys General Hospital, Passiac, USA; 3 Gastroenterology, New York Medical College at Saint Michael's Medical Center, Newark, USA

**Keywords:** gastrointestinal oncology, gastro-intestinal surgery, tumor-lysis syndrome, cancer immunotherapy, metastatic gastric adenocarcinoma

## Abstract

Tumor lysis syndrome (TLS) emerges as a critical oncological emergency, a consequence of the body's struggle to manage the intense cellular turnover and release of cytotoxins induced by treatments such as chemotherapy, radiation, targeted immune therapy, or hormonal therapy. While commonly associated with hematological malignancies, the heightened risk also extends to advanced-stage solid tumors and instances of liver metastasis. Although TLS is a rare occurrence in gastric adenocarcinoma, reported cases are usually linked to the initiation of chemotherapy. Remarkably, the incidence of TLS following the commencement of pembrolizumab in gastric adenocarcinoma remains undocumented in the existing literature. In this context, we present a compelling case involving a 73-year-old gentleman diagnosed with advanced-stage metastatic gastric adenocarcinoma. Strikingly, the patient developed TLS subsequent to the initiation of pembrolizumab (Keytruda®). This unique scenario not only accentuates the atypical manifestation of TLS in the context of gastric adenocarcinoma but also underscores the need for heightened awareness and exploration of potential complications associated with immunotherapeutic agents in solid tumor settings. The detailed analysis of this case contributes valuable insights that may prove instrumental in refining our understanding of the intricate interplay between immunotherapy and tumor lysis syndrome in the specific landscape of gastric adenocarcinoma.

## Introduction

Tumor lysis syndrome (TLS) stands as a formidable oncological emergency, traditionally linked to hematological malignancies and solid tumors emerging after the commencement of chemotherapy [1]. The intriguing aspect of TLS in the context of gastric adenocarcinoma and its association with pembrolizumab, however, remains uncharted territory in medical literature [2]. This case report seeks to shed light on a distinctive instance involving a 73-year-old male grappling with advanced-stage metastatic gastric adenocarcinoma. Remarkably, TLS surfaced as a critical concern shortly after the initiation of treatment with pembrolizumab. Unraveling the intricacies of this atypical manifestation could potentially contribute to a deeper understanding of the interplay between immunotherapy and TLS in the context of solid tumors, presenting a crucial addition to the current body of clinical knowledge.

## Case presentation

A 73-year-old male with a history of hypertension and metastatic gastric cancer involving the liver, lymph nodes, and bones was diagnosed via endoscopy with gastric biopsy nine months ago. He was diagnosed with gastric cancer nine months prior to his presentation, starting treatment one month late with two cycles of FOLFOX (the chemotherapy regimen made up of the drugs folinic acid (FOL), fluorouracil (F), and oxaliplatin (OX)) followed by XELOX (capecitabine plus oxaliplatin). He continued maintenance therapy with capecitabine (Xeloda). Two months prior to his presentation, a repeat CT scan showed extensive disease progression, including liver, lymph node, and bone metastasis, along with elevated CEA markers. He was found to have advanced programmed death ligand 1-positive (combined positive score of 10). This prompted a switch to targeted therapy with pembrolizumab (Keytruda®). Six days after pembrolizumab initiation, the patient met both laboratory TLS (L-TLS) and clinical TLS (C-TLS) criteria. He presented to the emergency department with progressively worsening confusion, restlessness, weakness, nausea, and non-bloody vomiting over the last six days, following chemotherapy treatment one week earlier. He had recently initiated pembrolizumab and experienced a drop in hemoglobin to 7.6 g/dL normal range (12-15.5 g/dL), necessitating a one-unit packed red blood cell transfusion two weeks prior to his presentation. his laboratory results before starting the medication (Table 1). A review of systems revealed significant weight loss over the last nine months, decreased appetite, abdominal pain, and intermittent constipation. On physical examination, he was afebrile but hypotensive (74/36 mmHg), fatigued but alert and oriented, with scleral icterus and jaundice.

**Table 1:** Laboratory results on the presentation day BUN: Blood urea nitrogen; ALT: Alanine aminotransferase; AST: Aspartate aminotransferase

	Laboratory results before starting the medication	Laboratory results on admission	Normal range
Creatinine	1.4 mg/dl	8.8 mg/dl	0.5-1.0 mg/dl
BUN	30 mg/dl	>150 mg/dl	6-24 mg/dl
Potassium	4.7 mmol/L	7.1 mmol/L	3.5-5.3 mmol/L
phosphorous		10.1 mg/dl	2.1-4.3 mg/dl
Uric acid		26 mg/dl	4.0-8.0 mg/dl
Calcium	8.4 mg/dl	7.8 mg/dl	8.6-10.4 mg/dl
WBC	24.80 10*3/ul	23.10 10*3/ul	4.4-11.0 10*3/ul
Hemoglobin	10.1 gdl	9.1 g/dl	12-15.5 g/dl
ALT	75 U/L	165 U/L	6-29 U/L
AST	208 U/L	791 U/L	10-36 U/L
T.Bilirubin	6.2 mg/dl	20.2 mg/dl	02-1.2 mg/dl
Alkaline phosphatase	337 U/L	347 U/L	33-130 U/L
Lipase		936 U/L	12-53 U/L
Lactic acid		4.9 mmol/L	0-2 mmol/L

**Figure 1:**
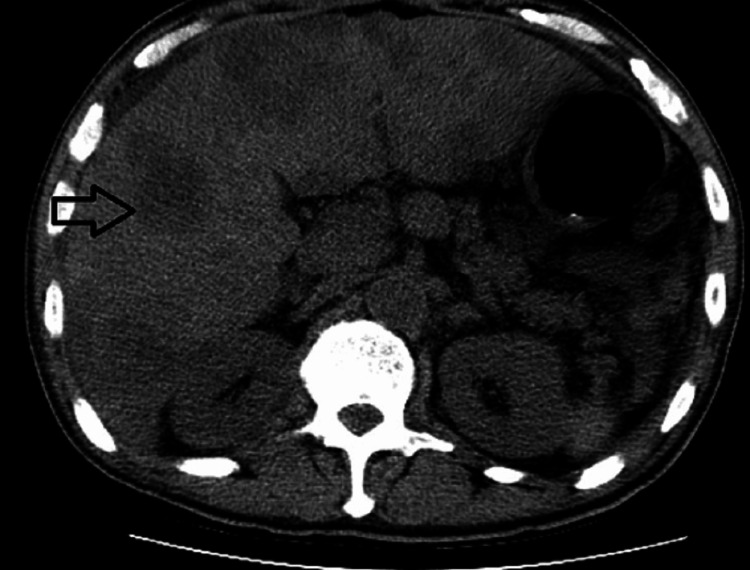
CT abdomen without contrast CT abdomen without contrast revealed hepatomegaly with multiple hypodense masses representing metastatic disease (black arrow).

Urinalysis indicated dark yellow, turbid urine, positive for nitrates, bilirubin, a few white blood cells and red blood cells, and >30 bacteria. Chest X-ray showed no acute cardiopulmonary process. Computerized tomography (CT) of the abdomen and pelvis showed hepatomegaly with multifocal masses and ascites (Figure 1). Abdominal ultrasound revealed diffuse extensive hepatic masses, a decompressed gallbladder with marked wall thickening but no bile duct dilation, and small perihepatic ascites.

The patient was transferred to the intensive care unit (ICU), where he received fluids, vasopressors including norepinephrine and vasopressin for hypotension, daptomycin and meropenem for a possible intra-abdominal infection, and underwent Shiley catheter placement followed by hemodialysis for worsening renal function. Gastroenterology was consulted for hyperbilirubinemia, likely due to intrahepatic bile duct obstruction from metastatic gastric cancer to the liver, with no bile duct dilation. Active intervention was postponed due to the patient's hemodynamic instability. Paracentesis was recommended but deferred for the same reason. Further laboratory results were reactive for Hepatitis C antibodies. Urine and blood cultures were negative, and daptomycin was discontinued. During this admission, the patient's mental status continued to fluctuate due to acute metabolic encephalopathy, and hypotension worsened despite increasing medication. Goals of care discussions with the family led to the decision to transition the patient to inpatient hospice care. After one week of admission, the patient was taken off pressors and passed away.

## Discussion

Tumor lysis syndrome (TLS) is a critical oncological emergency that can occur spontaneously or following the initiation of treatments such as chemotherapy, radiation, targeted immune therapy, or hormonal therapy [3]. TLS arises from the body's inability to manage the rapid cellular turnover and the release of cytotoxins into the bloodstream by tumor cells, leading to hyperuricemia, hyperkalemia, hyperphosphatemia, and hypocalcemia. Several risk factors, including age, tumor chemosensitivity, pre-existing conditions, and the patient's hydration status before therapy, increase the risk of TLS [4].

In accordance with the Cairo-Bishop classification, tumor lysis syndrome (TLS) is categorized into two main types: laboratory TLS (L-TLS) and clinical TLS (C-TLS) [5]. L-TLS is characterized by alterations in two or more quantifiable parameters, including uric acid levels reaching 8 mg/dL or higher, potassium levels at 6 mmol/L or greater, phosphorus levels of 2.1 mmol/L or more, calcium levels of 1.75 mmol/L or less, or a 25% change from the baseline in any of these electrolytes. On the other hand, C-TLS is identified when L-TLS is present alongside at least one clinical manifestation, such as renal insufficiency, arrhythmia, seizure, or sudden death [5]. This classification system provides a structured framework for the assessment and diagnosis of TLS, distinguishing between purely laboratory-based abnormalities and those accompanied by clinically significant complications.

TLS commonly occurs in hematologic cancers due to their high cell turnover, rapid proliferation rates, and increased chemosensitivity, with non-Hodgkin lymphoma and acute lymphoblastic leukemia being the most common. It is less common in solid tumors and very rarely occurs in gastric cancers [6]. Most cases of TLS in gastric cancer occur after starting chemotherapy [7]. The mortality for TLS stemming from solid tumors is moderately high at 35% compared to the 1.9% rate reported for patients with ALL and NHL [8].

Pembrolizumab is a monoclonal immunoglobulin (Ig)G4 kappa antibody that has been humanized and is specifically designed to target the programmed death receptor-1 (PD-1) receptor on the surface of human lymphocytes. The PD-1 receptor plays a crucial role as an "immune checkpoint," preventing the immune system from attacking the body itself. Some types of tumors exhibit high levels of programmed death receptor ligand-1 (PD-L1) expression. In other cases, certain tumor types employ adaptive immune resistance by leveraging the natural physiological process of PD-L1 induction, originally intended for protecting against immune-mediated damage from infections and redirecting it towards anti-tumor responses. When PD-L1 binds to PD-1, it leads to the inhibition of T-cell function. Pembrolizumab works by blocking the formation of the PD-1: PD-L1 complex, thereby enhancing T-cell-mediated killing [9]. Notably, pembrolizumab monotherapy has exhibited enduring antitumor efficacy in individuals with advanced programmed death ligand 1-positive (combined positive score ≥1) gastric/gastroesophageal junction adenocarcinoma [10]. Although the incidence of tumor lysis syndrome (TLS) following pembrolizumab is exceptionally rare, its occurrence is linked to a markedly unfavorable outcome [10]. It is noteworthy that, to date, the literature lacks any documented cases of TLS after initiating pembrolizumab in gastric adenocarcinoma. Therefore, our report represents a pioneering documentation of this complication following the commencement of immunotherapy in the context of gastric adenocarcinoma.

Initial treatment involved intravenous (IV) hydration, IV thiamine, and chemical dialysis to address acute kidney injury (AKI) and hyperkalemia [11]. Patients receiving oncological treatment should undergo evaluation before, during, and after treatment initiation to assess TLS risk. Risk stratification involves criteria for L-TLS to classify patients into low, intermediate, and high-risk categories. Solid tumors, especially in advanced stages with metastasis, can elevate TLS risk. Patient education regarding C-TLS symptoms is crucial upon chemotherapy initiation to ensure timely medical attention [12]. In addition, as TLS can occur with immunotherapy, we suggest initiating preventive measures such as hydration,, keeping the urine output > 100 ml/24h before starting the treatment.

## Conclusions

Tumor lysis syndrome (TLS) is a severe and potentially life-threatening condition that typically occurs in hematological malignancies and solid tumors post-chemotherapy. This report highlights an exceptional case of TLS in a patient with advanced-stage metastatic gastric adenocarcinoma following the initiation of pembrolizumab. Although the frequency of TLS with pembrolizumab may be low, when it does occur, the mortality rate is high. Therefore, TLS should also be considered when using immune checkpoint inhibitors. We are bringing attention to this case to emphasize that TLS can occur with immunotherapy, albeit infrequently. We recommend initiating preventive measures before commencing immunotherapy and promptly starting treatment if TLS occurs.
